# Production of Xylanase by *Trichoderma* Species Growing on Olive Mill Pomace and Barley Bran in a Packed-Bed Bioreactor

**DOI:** 10.3390/jof10010049

**Published:** 2024-01-05

**Authors:** Kholoud M. Alananbeh, Rana Alkfoof, Riyadh Muhaidat, Muhannad Massadeh

**Affiliations:** 1 Department of Plant Protection, School of Agriculture, The University of Jordan, Amman 11942, Jordan; 2 Department of Biological Sciences, Faculty of Science, Yarmouk University, Irbid P.O. Box 21163, Jordan; kfoofrana@yahoo.com (R.A.); muhaidat@yu.edu.jo (R.M.); 3 Department of Biology and Biotechnology, Faculty of Science, The Hashemite University, Zarqa P.O. Box 11315, Jordan

**Keywords:** barley bran, olive mill pomace, packed-bed bioreactor, partial purification, solid-state fermentation, *Trichoderma* spp., xylanase

## Abstract

Xylanases are hydrolytic enzymes that have tremendous applications in different sectors of life, but the high cost of their production has limited their use. One solution to reduce costs and enhance xylanase production is the use of agro-wastes as a substrate in fungal cultures. In this study, olive mill pomace (OMP) and barley bran (BB) were used as carbon sources and possible inducers of xylanase production by three species of *Trichoderma* (*atroviride*, *harzianum*, and *longibrachiatum*), one major xylanase producer. The experiments were conducted under a solid-state fermentation system (SSF) in flask cultures and a packed-bed bioreactor. Cultures of OMP and BB were optimized by examining different ratios of OMP and BB, varied particle sizes, and inoculum size for the three species of *Trichoderma*. The ratio of 8:2 OMP and BB yielded the highest xylanase activity, with a particle size of 1 mm at 29 °C and an inoculum size of 1 × 10^7^ spores/mL. Studying the time profile of the process revealed that xylanase activity was highest after seven days of incubation in flask SSF cultures (1.779 U/mL) and after three days in a packed-bed bioreactor (1.828 U/mL). The maximum percentage of OMP degradation recorded was about 15% in the cultures of *T. harzianum* flask SSF cultures, compared to about 11% in *T. longibrachiatum* bioreactor cultures. Ammonium sulfate precipitation and dialysis experiments showed that Xylane enzyme activity ranged from 0.274 U/mL in *T. harzianum* to 0.837 U/mL in *T. atroviride* when crude extract was used, with the highest activity (0.628 U/mL) at 60% saturation. Xylose was the main sugar released in all purified fractions, with the G-50 and G-75 fractions showing the maximum units of xylanase.

## 1. Introduction

There is a growing interest in screening natural sources to find bioactive compounds with nutraceutical and industrial benefits [1]. Increasing synthetic costs and environmental concerns rationalize the search for natural, high-value products to produce different commodities [2]. Therefore, attention has been drawn to plant biomass and agricultural wastes (agro-industrial residues) because they are cheap and abundant around the world, and major research efforts are underway to use them from different perspectives [3]. The agro-residues are primary reservoirs of lignocellulose, rendering them particularly appealing for many products essential to human society [4].

Xylan, a major hemicellulosic constituent of lignocellulosic materials, is markedly valued from industrial and biomedical perspectives and for the production of value-added products [5,6]. It is the most abundant hemicellulose in plant cell walls, accounting for more than 30% of the plant biomass [4,7,8]. Appreciable proportions of xylan exist in hardwoods and softwoods, as well as herbaceous plants, and form up to 35% of the lignocellulosic materials [9].

Xylan has a backbone of β-1,4-D-xylose residues with different α-glycosidically linked substitutions in side chains, primarily including L-arabinofuranose, D-galactose, and D-glucuronic or 4-O-methyl-d-glucuronic acid [10,11]. Substituents such as acetyl, arabinosyl, glucuronysyl, coumaroyl, and ferulic acid esters can also be found. The proportion of these substituents and the degree of branching are variable, depending on the plant source [12]. Complete degradation of xylan is achieved by a set of hydrolytic enzymes collectively called xylanases or xylanolytic enzymes [13]. These enzymes have been the target of many research endeavors; yet they are expensive and required in large amounts to be recruited in food, industrial, and medical applications. For this reason, attention has been paid to the natural producers of xylanases and agro-industrial wastes to develop nutrient-rich media for xylanase-producing organisms.

Xylanases find extensive applications across various industries, including food and feed, paper and pulp, textiles, pharmaceuticals, and lignocellulosic biorefinery [9]. Xylanases are ubiquitous and produced by a variety of organisms, including bacteria, protozoa, algae, fungi, snails, crustaceans, and insects [9,10,14]. Among these, multicellular fungi have been highly acknowledged as tremendous producers of xylanases [9,15]. Their filamentous structure provides large surface areas for absorptive nutrition and enhanced production of xylanases [2,16]. Species of the genus *Trichoderma* such as *T*. *reesi*, *T*. *viride*, and *T*. *harzianum* have held a key position in research studies aiming at xylanase production and purification [9,17].

Olive mill pomace (OMP) is one of the most cost-effective and nutrient-rich substrates used for cultivating fungi. Large quantities of OMP are produced in the Mediterranean region. The extraction of olive oil yields ca. 15,000 tons of olive oil and 80,000 tons of OMP a year [18]. In recent years, Jordan has been the eighth largest producer of olive oil worldwide, with 24,000 tons of oil and 60,000 tons of OMP per year [19]. Traditionally, OMP is used as firewood, but this causes air pollution. Consequently, the authorities placed restrictions on olive oil extraction factories in terms of waste accumulation and re-use. One solution is to make use of OMP as a growth medium for many microorganisms in attempts to produce commercial and high-value products (i.e., enzymes, bio-control agents, fertilizers, and biofuel), and interest in this is increasing [20]. However, few studies exploited OMP to produce enzymes and other value-added products.

The present study aims to examine the capacity of selected *Trichoderma* species isolated from plant sources for xylanase production using solid-state fermentation (SSF) and varied combinations of OMP and barely bran (BB) substrates, to determine optimal growth conditions for xylanase production, and to extract and partially purify xylanase. Despite the many studies on xylanase production from *Trichoderma* spp. around the world, this study is, to our knowledge, the first on Jordanian isolates of *Trichoderma*. The process of SSF has been accredited for the production of value-added microbial enzymes using agro-industrial origins. It is highly productive, with higher product stability and lower processing costs compared to the submerged fermentation process [21].

## 2. Materials and Methods

### 2.1. Fungal Species Isolates and Inoculum Preparation

Isolates of three distinct *Trichoderma* species (*T*. *atroviride* [accession MT626716], *T*. *harzianum* [accession MT626717], and *T*. *longibrachiatum* [accession MT626720]) were examined for xylanase production. The isolates were sub-cultured on potato dextrose agar (PDA) growth medium, incubated for 5 days at 28 °C, and used for inoculum preparation.

The inoculum of each isolate was prepared by adding 1 mL of sterile distilled water to a fresh culture of PDA plate, swept with a loop, and filtered to produce a slurry. After that, the suspension was transferred to 99 mL of sterile distilled water. The spores were counted using a hemocytometer to obtain a spore concentration of (10^7^ spores/mL), and the suspension was stored in the refrigerator at 4 °C until use [22].

### 2.2. Preparation of OMP

Freshly harvested OMP was collected from olive mills located in Zarqa city (Jordan) during the period of olive oil extraction (October–December 2019). The collected pomace was dried in the greenhouse and used as the main substrate in culturing and fermentation processes.

### 2.3. Flask Solid State Fermentation for Xylanase Production

This part of the study was carried out following [8,23]. For each *Trichoderma* sp., a flask containing a 10 g mixture of OMP and barley bran (BB) was autoclaved at 121 °C for 15 min. Then, the mixture was moistened with 5 mL of sterile distilled water and inoculated with a spore suspension of each *Trichoderma* species (10^7^ spores/mL). All flasks were incubated for seven days at 29 °C until further use in further biochemical analyses.

### 2.4. Xylanase Assay

The extraction and assaying of xylanase were carried out according to Al Sheikh [24], Assamoi et al. [25], and Mardawati et al. [8]. The contents of each prepared flask were collected in new flasks, and 50 mM sodium citrate solution (pH 5) was added. Then, the flasks were rotated at 150 rpm at room temperature for 30 min on an orbital shaker (Human Lab, Seoul, Republic of Korea) and filtered through Whatman No. 1 filter paper. The filtrate was centrifuged at 6000 rpm (Wagtek, Thatcham, UK) for 15 min at 4 °C, and the supernatant was used for the xylanase assay. Xylanase activity was measured by adding 0.2 mL of the sample supernatant to 0.5 mL of 1% oat-spelt xylan solution in 0.3 mL of 50 mM citrate buffer (pH 5) at 45 °C for 30 min. Reducing sugars released by xylanase were determined using 3 mL of 3.5-dinitrosalicylic acid (DNS) at 100 °C for 5 min according to the DNS method [15,26,27]. The amount of reducing sugars released in the reactions was measured spectrophotometrically at 540 nm. Xylanase activity was calculated based on a standard curve of serial concentrations of xylose. One unit (U) of xylanase activity was defined as the amount of enzyme releasing one µmole of reducing sugar (xylose equivalent) per min under the assay conditions [8,24].

### 2.5. Assessment of Reducing Sugars

Reducing sugar released by each culture was assessed using the Nelson–Somogyi methodology [28]. One milliliter of Somogyi reagent was added to 1 mL of the sample supernatant. The mixture was vortexed and boiled for 15 min at 100 °C. Next, 1 mL of Nelson reagent was added, and the absorbance was measured at 520 nm. Concentrations of reducing sugars were determined based on a standard curve of xylose concentrations and following the methodology of [29].

### 2.6. Total Carbohydrates

The total carbohydrates of each culture were analyzed by the phenol–sulfuric acid method and detected photometrically [30]. Fifty microliters of 80% phenol solution was added to 50 µL of sample supernatant, then 2 mL of sulfuric acid were added. The absorbance was read at 490 nm after 10 min of reaction progress. Concentrations of the total carbohydrates for each culture were estimated using a standard curve.

### 2.7. Optimization of Xylanase Production by Trichoderma *sp*.

Different ratios of OMP and BB (9.5:0.5, 9:1, and 8:2) and of different supplement particle sizes of OMP and BB (5 mm, 2 mm, and 1 mm) were prepared to determine the best combination that enhances growth of *Trichoderma* species and xylanase production. Test trials were conducted in flasks containing trace elements of a nitrogen source (ammonium sulfate or peptone) and a carbon source (xylose or xylan) at a concentration of 1% of the culture medium [31]. A culture containing only OMP was included as a control. The mixture of 8:2 OMP and BB (8 and 2 g/10 mL of distilled water) with particle sizes of 1 mm supplied with xylan (1% of medium) and peptone (1% of medium) yielded the highest growth and so was chosen as the culture medium in the following packed-bed column SSF.

### 2.8. Packed-Bed Column Solid State Fermentation of OMP and BB for Xylanase Production

This experiment was conducted following the method of [32]. Cultures yielding optimal growth from the flask fermentation were transferred into a sterile packed-bed bioreactor composed of a double-jacketed glass column (50 × 5 cm) connected to an air filter pump via silica tubes supplied through a 0.22 µm pore size filter at the bottom with a 20–25 air bubble flow rate that is controlled by a flow meter. The bioreactor was packed with multilayers of sterile stainless-steel mesh, and the culture medium was moistened with sterile distilled water and mixed with a spore suspension of *Trichoderma* species (10^7^ spores/mL). The column was then incubated at 29 °C for 3 days and supplied with air at 1 vvm. Finally, the fermented substrate was used for the different biochemical analyses employed in the abovementioned flask SSF. This experiment was repeated three times.

### 2.9. Percentage of Degradation

To determine the percentage of the substrate consumed by *Trichoderma* sp., the fermented culture medium was weighed (initial weight) and then dried at 50 °C for 24 h and weighed (final weight). The degradation percentage was calculated using the following equation:

(Initial weight − Final weight)/Initial weight × 100%

### 2.10. Times Profile of the Process

To determine the optimal incubation time for the cultures of the three *Trichoderma* isolates, two flask SSF replicates of each species were mixed with its spore suspension (10^7^ spores/mL) and incubated at 29 °C for five days. Afterwards, they were filtered daily, and the filtrate was used for biochemical assays of xylanase activity, total carbohydrates, reducing sugars, and percentages of degradation.

### 2.11. Partial Purification of Xylanase

#### 2.11.1. Ammonium Sulfate Precipitation and Dialysis

Crude xylanase extract was subjected to a graded ammonium sulfate saturation (20 to 70%) with continuous stirring on ice. Samples were then centrifuged at 5000 rpm for 15 min. For each percentage, the pellet formed was dissolved in a minimum amount of 50 Mm citrate buffer (pH 5), and enzyme activity and protein concentration were measured. The sample with the highest xylanase activity was further processed using dialysis bags in citrate buffer for 24 h at 4 °C. Afterwards, xylanase activities and protein concentrations were re-measured [33,34].

#### 2.11.2. Size-Exclusion Chromatography

After ammonium sulfate precipitation and desalting of proteins, the highest protein concentration of dialyzed samples was subjected to a column (diameter: 1.5 cm, length: 30 cm, Bio-Rad, Hercules, CA, USA) containing Sephadex G-75 as the stationary phase, and in another trial, the column contained Sephadex G-50. The column was eluted with citrate buffer, pH 5. The eluted solution was collected in Cuvette tubes as fractions, each of 1 mL in size. Each fraction was used to measure dissolved protein concentration at 280 nm, enzyme activity in U/mL, and specific activity in U/mg protein as previously described [35,36].

### 2.12. Statistical Analysis

Standard curves of xylanase enzyme activity, reducing sugar, total carbohydrates, and protein concentration were drawn using Microsoft Excel 2010. One-way analysis of variance (ANOVA) was used for different testing among the experimental groups. Data were calculated as the mean ± standard error of the mean (SE) for duplicate independent experiments, and *p* ≤ 0.05 was considered a significant difference.

## 3. Results

### 3.1. Flask Solid State Fermentation (SSF) for Xylanase Production by Trichoderma *sp*.

Three *Trichoderma* species (*T*. *atroviride* MT626716, *T*. *harzianum* MT626717, and *T*. *longibrachiatum* MT626720) were tested for xylanase production in flask SSF. Their optimum growth temperature was examined using PDA agar plates. The optimum temperature for their growth was 29–30 °C, where maximum biomass concentration was observed. The three species entered the stationary phase, as indicated by spore formation, after five days of incubation.

**Figure 1:**
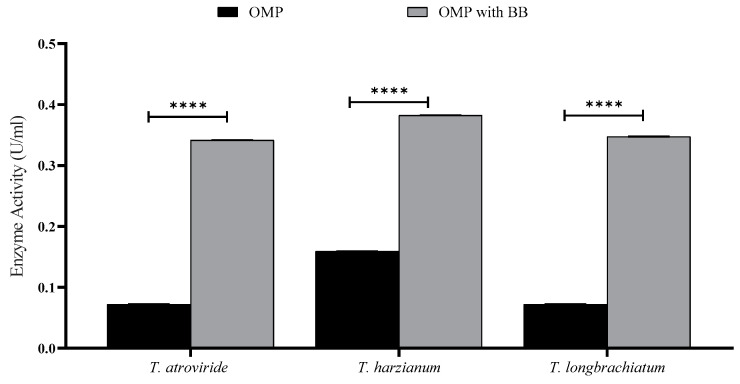
Xylanase activity of *T. atroviride*, *T. harzianum*, and *T. longibrachiatum* cultured on OMP with and without BB after seven days of fermentation. Bars represent the means (±SE) of two replicates for each culture. Asterisks indicate statistically significant differences at *p* ≤ 0.05. **** *p* < 0.0001.

To perform SSF for enzyme production, the three *Trichoderma* spp. were grown on OMP with or without BB under static conditions without any supplements. It was noticed that fungal mycelia penetrated through the substrate particles of the culture after three days of incubation, and sporulation started to appear afterward. Xylanase activity, reducing sugar, and total carbohydrate concentrations were measured for seven-day-old *Trichoderma* spp. cultures (Figure 1). The data illustrate that supplementing OMP with BB enhanced enzyme production and fungal growth as well. The three fungal species showed almost the same behavior in producing the xylanase enzyme (Figure 1).

*T. atroviride* was able to liberate 43.725 μg/mL of reducing sugars compared to 22.835 mg/mL in the culture of *T. longibrachiatum*. On the other hand, the maximum total carbohydrate concentration obtained was from the cultures of *T. longibrachiatum* (1.92 μg/mL). Based on these results, combination cultures of OMP and BB were used for further studies.

### 3.2. Optimization of SSF for Xylanase Production

**Figure 2:**
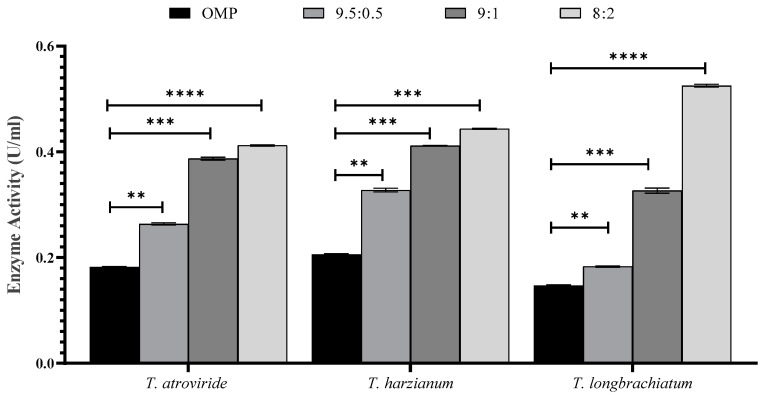
The effect of the culture ratio between OMP and BB on xylanase activity in the three studied *Trichoderma* species after seven days of fermentation. Bars represent the means (±SE) of two replicates for each culture. Asterisks indicate statistically significant differences at *p* ≤ 0.05. ** *p* < 0.01, *** *p* < 0.001, and **** *p* < 0.0001.

**Figure 3:**
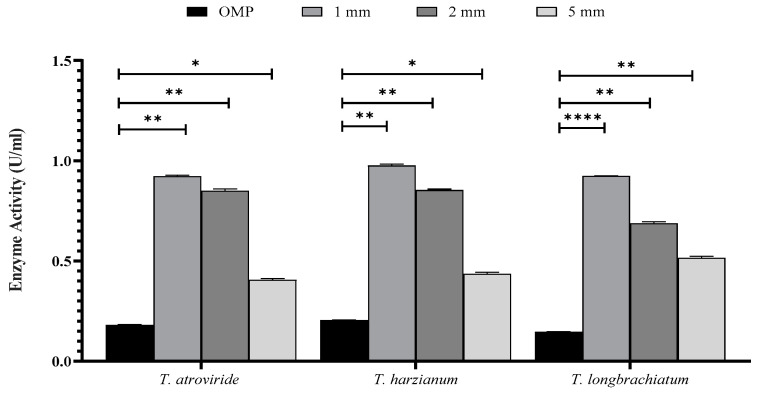
The effect of particle size of OMP and BB on xylanase activity in the three studied *Trichoderma* species after seven days of fermentation. Bars represent the means (±SE) of two replicates for each culture. Asterisks indicate statistically significant differences at *p* ≤ 0.05. * *p* < 0.05, ** *p* < 0.01, and **** *p* < 0.0001.

The effect of OMP and BB mixing ratios and sizes, in addition to the supplementation of cultures with trace elements, was studied. The results revealed a significant difference in the activity of xylanase and concentrations of reducing sugars and total carbohydrates in the three cultures of *Trichoderma* sp. compared to that of the control (*p* < 0.001; Figure 2 and Figure 3).

The activity of xylanase was significantly higher in all ratios and sizes tested compared to that of the control. It was noticed that there were variations in xylanase activity within each *Trichoderma* sp. culture (Figure 2 and Figure 3). The ratios of 8:2 and 9.5:0.5 of OMP and BB gave the highest and lowest xylanase activity, respectively (Figure 2). In Figure 3, the particle size of 1 mm was found to be the most effective in xylanase production compared to 2 mm and 5 mm in all *Trichoderma* species studied. Similarly, the inclusion of trace elements in growth cultures exerted a significant effect on enhancing *Trichoderma* to produce more active xylanase (Figure 3).

**Figure 4:**
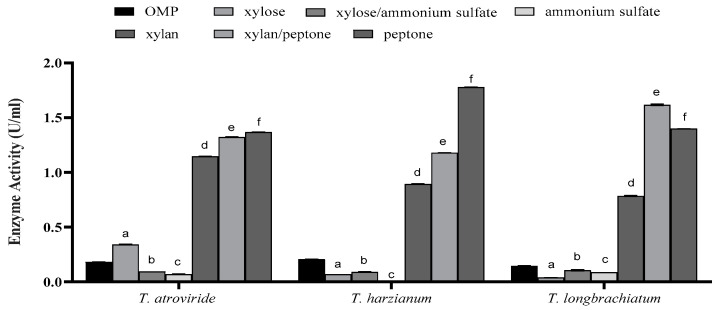
The effect of supplementation of growth cultures with trace elements on xylanase activity for the three studied *Trichoderma* species after seven days of fermentation. Bars represent the means (±SE) of two replicates for each culture. Different letters indicate statistically significant differences at *p* ≤ 0.05.

However, the effect was dependent on the type of supplement included in the culture. In all species, supplementation with xylan, peptone, or a combination of both yielded higher xylanase activity compared to all other types of supplements and the controls (Figure 4). Notably, peptone alone was found to be the most effective in xylanase production in all *Trichoderma* species, irrespective of the presence or absence of xylan and despite the overall variations observed (Figure 4).

### 3.3. Time Profile of the Process

**Figure 5:**
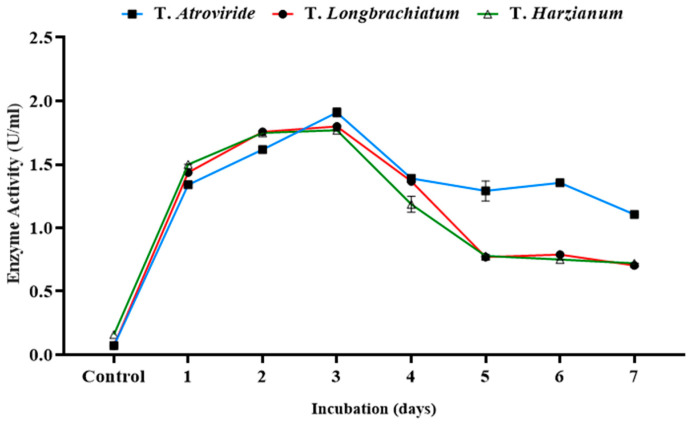
Xylanase activity over a seven-day incubation period in three studied *Trichoderma* species incubated at 29 °C.

The time profile for the production process was studied in the three tested cultures of *Trichoderma* sp. at the optimum conditions determined previously. The xylanase activity, reducing sugar, and total carbohydrate concentrations were measured independently every day over the incubation period (Figure 5). Over the incubation period, the production of the xylanase enzyme for the three tested species of *Trichoderma* reached its maximum on day three. Therefore, three-day-old cultures were chosen for further studies.

### 3.4. Packed-Bed Column Solid State Fermentation

**Figure 6:**
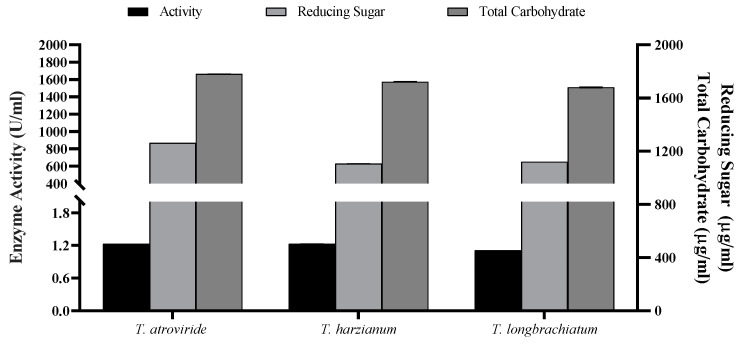
Xylanase activity and concentrations of reducing sugars and total carbohydrate of the three *Trichoderma* cultured species using packed-bed bioreactor.

**Figure 7:**
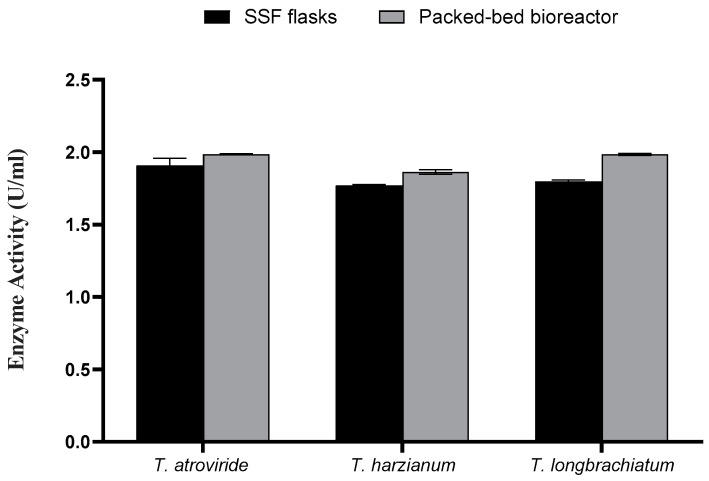
Xylanase activity using SSF and packed-bed bioreactor.

The data of the flask SSF process for xylanase enzyme production were translated into a column bioreactor packed with OMP and BB under SSF conditions. After three days of fermentation, all measures were significantly higher compared to those achieved from flask SSF cultures (Figure 6 and Figure 7). For instance, the maximum xylanase activity achieved was 1.998 U/mL for *T. harizianum* (Figure 7). The maximum total reducing sugars (867.08 mg/mL) and total carbohydrate (1.667 mg/mL) were the highest in *T. atroviride* compared to the other two species.

### 3.5. Percentage of OMP Degradation

**Figure 8:**
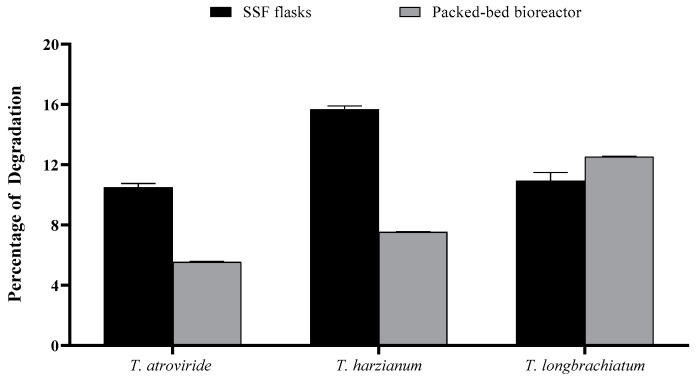
Consumed and percentage of culture media after seven days of incubation using SSF and packed-bed bioreactor by the three *Trichoderma* species studied. Bars represent the means (±SE) of four replicates per species.

The percentage of substrate degradation varied between SSF in flasks and in the packed-bed bioreactor, with the former being generally greater than the latter except for *T. longibrachiatum*. Using the SSF, the percentage varied in the following order: *T. harzianum* > *T. longibrachiatum* > *T. atroviride* (Figure 8). The maximum percentage of degradation recorded was about 15% in the cultures of *T. harzianum* flask SSF cultures, compared to about 11% in *T. longibrachiatum* bioreactor cultures.

### 3.6. Partial Purification of Xylanase

The crude extract of each *Trichoderma* species that gave the highest xylanase activity using a packed-bed bioreactor was partially purified through a series of ammonium sulfate precipitation reactions and dialysis. The highest dissolved protein concentration was obtained using a 60% ammonium sulfate saturation, and this percentage was chosen for further purification of xylanase using size-exclusion chromatography.

**Figure 9:**
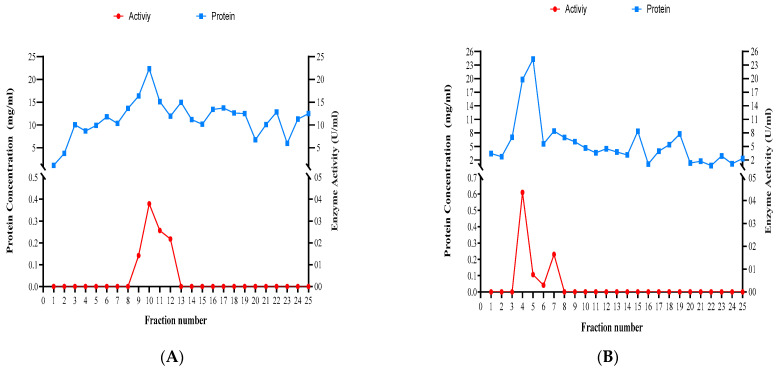

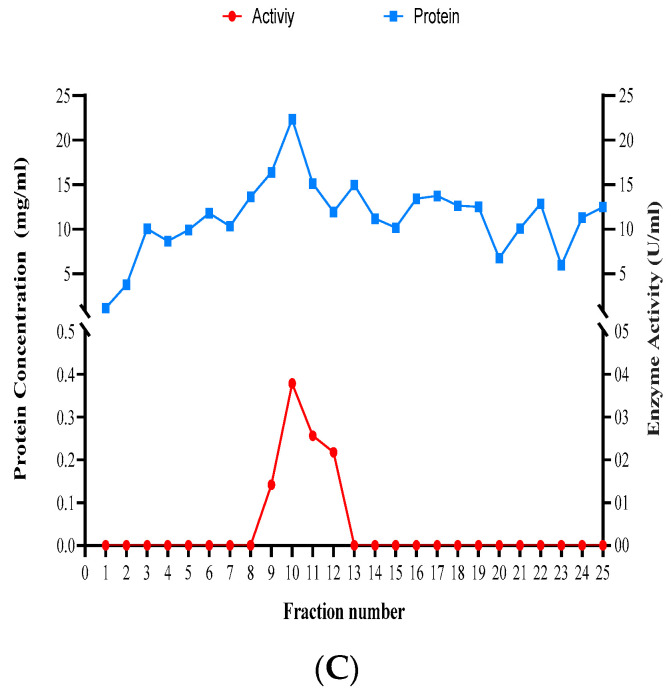
Concentrations of dissolved proteins and xylanase activity in the fractions obtained using size-exclusion chromatography for the three studied *Trichoderma* species. (**A**) *T. atroviride*, (**B**) *T. harzianum*, and (**C**) *T. longibrachiatum*.

The experiment of size-exclusion chromatography yielded 25 fractions per *Trichoderma* species tested (Figure 9). Dissolved proteins and xylanase activity were highest in fractions 9 to 12 for *T. atroviride*, 4 to 7 for *T. harzianum*, and 9 to 14 for *T. longibrachiatum* (Figure 9).

Xylanase enzymatic activity ranged from 0.274 U/mL in *T. harzianum* to 0.837 U/mL in *T. atroviride* when crude extract was used. In general, xylanase activity was the highest in *T. atroviride* compared to the other two species across all the ammonium sulfate precipitation concentrations (20–70%). In addition, the specific activity of xylanase was measured for the three tested species of *Trichoderma*, and data ranging from 0.001 to 0.064 U/mg for *T. atroviride*, 0.004 to 0.031 mg/U for *T. harzianum*, and from 0.003 to 0.057 for *T. longibrachiatum* were obtained (Appendix A).

## 4. Discussion

In this study, a combination of olive mill pomace (OMP) and barley bran (BB) was studied as substrates for the production of the xylanase enzyme by *Trichoderma* sp. Different lignocellulosic materials were used as substrates to produce xylanase, and several reports illustrated the ability of *Trichoderma* species to use lignocellulosic materials as nutrient sources [37]. The agro-industrial residues of OMP have been well recognized in the literature as a rich source of organic matter, comprising a large amount of cellulose, hemicellulose, lignin, lipids, carbohydrates, and phenols [38]. This complex structure makes it suitable for the growth and metabolism of many microorganisms, specifically *Trichoderma* [39]. However, there are limited studies in the literature on using a combination of OMP and BB as an enzyme inducer to produce xylanase.

The activity of xylanase was studied with and without BB throughout the incubation period. The highest activity of xylanase was recorded with BB, and the incubation period was seven days. This indicates that the BB used in this study supports the growth of *Trichoderma* spp. and enhances their capacity to produce xylanase, an inducible enzyme. This is consistent with the findings of Soliman [40], who underscored BB as an excellent nutritive material that stimulates *Trichoderma* to produce xylanases because its hemicellulosic content is composed largely of xylan [40]. The substrates OMP and BB are thus vital for enhancing gene expression and protein synthesis, and, in consequence, the activity of cells to produce xylanase is augmented. It is well accepted that when cells are in the stationary phase of growth, their priority to express a specific gene varies depending on their needs and the signals they receive from their environment.

Xylanase activity increased after the inclusion of OMP and BB at a ratio of 8:2 and a particle size of 1 mm. It was necessary to reduce the substrate particle size, which may provide a favorable surface area for fungal growth, uptake of gases, accessibility, and hence improved production of xylanase. Lakshmi [41] affirmed that a smaller substrate particle size provides more efficient nutrient uptake, better support for microbial attachment, and better transport of substrate components compared to larger particle sizes.

To further enhance fungal growth and enzyme production, 1% xylan and peptone were added to the cultures as additional carbon and nitrogen sources, respectively. This supplement increases xylanase activity. Xylan was added as a simple inducer of gene expression and xylanase synthesis, whereas peptone was utilized by *Trichoderma* as a protein source. Once a high fungal biomass is established, *Trichoderma* can achieve complete hydrolysis of xylan. Therefore, the addition of 1% of these constituents to the fermentation media helps the microorganism attain its maximum xylanase production capability in a shorter period of time owing to the easily utilized carbon and nitrogen sources in the media. Our results support those of Gauterio et al. [42], who maximized xylanase production by *Aureobasidium pullulans* using a by-product of rice grain supplied with xylan, peptone, and other nutritional sources. Moreover, a considerable increase in xylanase activity by *T. harzianum* 1073 D3 was reported when 1% xylose was included, surpassing the effects observed using alternative sugars such as glucose, galactose, fructose, lactose, and sucrose [43]. In a study by El-Gendi et al. [44] on xylanase production by *Bacillus subtilis* using varying peptone concentrations, a direct correlation between peptone concentration and xylanase production and strain growth was reported. The highest growth and productivity were found at a peptone concentration of 1.4%. Taking these findings as a baseline, peptone emerged as a primary organic nitrogen source required to enhance xylanase production and activity by bacterial and fungal species [43].

In this study, examining the temporal profile of the production process reveals that xylanase activity peaked after three days of incubation, followed by a sudden drop. This indicates that the fungal ability to generate the enzyme was strikingly restricted to the early phases of growth, allowing them to access available substrates necessary for their survival and reproduction. Subsequently, xylanase generation decreased during the transition between log and stationary phases of their normal growth cycles. From a commercial perspective, repeating solid-state fermentation (SSF) beyond a three-day incubation period would not yield significant results for xylanase production. The decline in nutrient availability and the build-up of metabolic by-products adversely impact fungal growth, pushing them into the stationary phase, where spore formation is induced. A similar trend of xylanase activity by *Trichoderma* using SSF was previously reported by Pandey [45].

To study xylanase enzyme properties, it was necessary to produce the enzyme in a larger quantity. For that reason, a packed-bed bioreactor was employed. Xylanase enzyme production was enhanced in the packed-bed bioreactor compared to the SSF flask due to the efficient aeration pattern in the packed-bed bioreactor, which provides air circulation in a closed system [45]. Also, a packed-bed bioreactor maintains adequate and homogeneous levels of temperature and moisture, allowing efficient transfer of nutrients and metabolites [46]. In a recent study by Massadeh et al. [47], the entrapment/immobilized fermentation method has been proposed as an alternative to classical free-cell fermentation owing to its prolonged periods of growth, enhanced metabolic activities, and repeated use of cells. Cell entrapment is a common methodology for whole-cell immobilization carried out in a polymer matrix, carrageenan and alginate, and synthetic fibers. As a result, fungal growth was greatly improved, xylanase activity increased, and the maximum percentage of substrate degradation was accomplished.

Partial purification of the xylanase enzyme was performed after protein precipitation with ammonium sulfate. The highest xylanase activity was achieved at 60% saturation of ammonium sulfate. The crude enzyme precipitate was dialyzed against solid sodium citrate, which caused a reduction in enzyme activity compared to the crude enzyme extract, which might be due to the high polarity of the protein that forced them to remain in their physical medium. This indicates that it is possible to separate proteins from a mixture on the basis of their relative hydrophilicity by gradually increasing the concentration of ammonium sulphate [42]. Gauterio et al. [42] reported a reduction in xylanase and cellulase activities when employing ammonium sulfate precipitation and dialysis against different buffer solutions.

The dialyzed samples with the highest activity were subjected to gel filtration chromatography using Sephadex G-50 and G-75. The results revealed that xylanase was partially purified, and its activity increased. This improvement could be due to the removal of other compounds affecting xylanase activity, as Sephadex G-50 and G-75 allowed the adhesion of proteins of specific sizes. Our results are in agreement with Silva et al. [48], who purified xylanase from *Trichoderma* sp. using Sephadex G-75 and claimed that the xylanase enzyme was purified [48].

Fractions showing the highest activity from the gel filtration chromatography were collected, and a sample of each fraction was spotted on silica gel plates to perform TLC. It was shown that xylose and glucose were the main sugars present in the enzyme extracts when compared to the standard employed (R. Alkfoof, K., Alananbeh, M. Massadeh and R. Muhaidat, unpublished results). Two distinct materials, namely OMP and BB, were employed as substrates for *Trichoderma* culture. The induction process triggered the enzymatic activity, leading to enzyme-mediated transformations. Upon conducting thin layer chromatography (TLC) post-enzyme purification, it was observed that OMP and BB exhibited high heterogeneity and intricate structural complexity. Their native structures posed challenges in comprehension. The enzymatic action on these materials resulted in not only xylose but also the formation of disaccharides and oligosaccharides. Given the endoxylanase nature of the enzyme, it acts on internal bonds within these structurally complex substrates. Unfortunately, due to the limited availability of disaccharides and oligosaccharides in the laboratory, a comprehensive experiment elucidating the complete spectrum of these materials was not conducted. Nevertheless, our analysis confirmed the presence of xylose in the reaction mixture. Our results agree with those of Silva et al. [48], who claimed that complete degradation of xylan releases xylose as the final product.

The xylanase enzyme has tremendous applications in industry, and it can be used in the synthesis of biofuels, foodstuffs, textiles, organic acids, and many value-added products. The agro-residues of OMP are cheap and available all through the year, and hence it is recommended to study its supplementation with different cost-effective elements to support the growth and metabolism of *Trichoderma*. To elucidate the fungal behavior in producing an active xylanase enzyme, it is recommended to investigate the type of xylanase produced by using SDS-PAGE, native PAGE electrophoresis, and HPLC for detecting the reaction products. In doing so, additional species of *Trichoderma* should be studied.

## 5. Conclusions

This study showed the ability of three *Trichoderma* species to produce xylanase by using OMP and BB as substrates in solid-state fermentation. After optimizing the growth of the three *Trichoderma* sp., xylanase enzyme production was assessed. It was found that BB supplementation was necessary to induce xylanase production. The optimum ratio of OMP and BB was 8:2, corresponding with the highest activity of xylanase recorded (0.527 U/mL) from the culture of *T*. *longibrachiatum*. Furthermore, using a 1 mm particle size of both substrates enhanced the activity of xylanase (0.983 U/mL) from the culture of *T*. *harzianum*. Supplementing the culture with xylan and peptone showed a positive impact on fungal growth and xylanase production in all *Trichoderma* sp. cultures. The maximum xylanase activity (1.944 U/mL) was obtained in the culture of *T*. *atroviride* after 3 days in SSF flask culture and in the packed-bed SSF bioreactor (1.998 U/mL). To extract the enzyme and partially purify it, the crude extract was collected from the packed-bed bioreactor culture of the three *Trichoderma* sp. and subjected to 60% ammonium sulfate precipitation and dialysis against solid sodium citrate. The dialyzed enzyme samples were introduced to size-exclusion chromatography using Sephadex G-75 and G-50. It was noticed that xylanase activity was concentrated, and to confirm its purity, the fractions were subjected to a TLC reaction. The results revealed that the selected fractions collected from the three *Trichoderma* sp. were highly active, liberating xylose as the main sugar observed.

## Data Availability

The data presented in this study are available on request from the corresponding author.

## Appendix A

Xylanase specific activity at different fractions for the three tested *Trichoderma* spp.

**Table:**

Fractions	*Trichoderma* species											
	*T. atroviride*				*T. harzianum*				*T. longibrachiatum*			
	OD_280_	Protein mg/mL	Activity U/mL	Specific Enzyme Activity U/mg	OD_280_	Protein mg/mL	Activity U/mL	Specific Enzyme Activity U/mg	OD_280_	Protein mg/mL	Activity U/mL	Specific Enzyme Activity U/mg
1	-0.035	-1.750	0.000	0.000	0.068	3.410	0.000	0.000	0.006	0.300	0.000	0.000
2	-0.030	-1.510	0.000	0.000	0.055	2.735	0.000	0.000	0.009	0.450	0.000	0.000
3	-0.001	-0.050	0.000	0.000	0.140	7.015	0.000	0.000	0.065	3.250	0.000	0.000
4	0.005	0.260	0.000	0.000	0.396	19.820	0.610	**0.031**	0.149	7.440	0.000	0.000
5	0.013	0.640	0.000	0.000	0.487	24.325	0.106	**0.004**	0.212	10.595	0.000	0.000
6	0.070	3.486	0.000	0.000	0.111	5.550	0.042	**0.007**	0.213	10.670	0.000	0.000
7	0.016	0.796	0.000	0.000	0.168	8.415	0.230	**0.027**	0.300	15.005	0.000	0.000
8	0.035	1.750	0.000	0.000	0.140	6.980	0.000	0.000	0.309	15.440	0.000	0.000
9	0.159	7.950	0.511	**0.064**	0.121	6.040	0.000	0.000	0.397	19.840	0.059	**0.003**
10	0.200	10.005	0.093	**0.009**	0.093	4.635	0.000	0.000	0.485	24.235	0.469	**0.019**
11	0.350	17.500	0.106	**0.006**	0.072	3.600	0.000	0.000	0.391	19.540	0.140	**0.007**
12	0.456	22.780	0.041	**0.002**	0.090	4.475	0.000	0.000	0.286	14.315	0.250	**0.017**
13	0.388	19.400	0.000	0.000	0.076	3.775	0.000	0.000	0.209	10.425	0.597	**0.057**
14	0.300	15.000	0.000	0.000	0.062	3.120	0.000	0.000	0.244	12.220	0.050	**0.004**
15	0.298	14.900	0.000	0.000	0.167	8.345	0.000	0.000	0.304	15.205	0.000	0.000
16	0.266	13.300	0.000	0.000	0.022	1.120	0.000	0.000	0.300	15.005	0.000	0.000
17	0.255	12.750	0.000	0.000	0.079	3.935	0.000	0.000	0.223	11.130	0.000	0.000
18	0.250	12.500	0.000	0.000	0.108	5.390	0.000	0.000	0.270	13.480	0.000	0.000
19	0.203	10.165	0.000	0.000	0.155	7.745	0.000	0.000	0.257	12.830	0.000	0.000
20	0.100	5.005	0.000	0.000	0.007	0.345	0.000	0.000	0.270	13.500	0.000	0.000
21	0.125	6.250	0.000	0.000	0.034	1.715	0.000	0.000	0.200	10.005	0.000	0.000
22	0.200	10.000	0.000	0.000	0.015	0.765	0.000	0.000	0.190	9.480	0.000	0.000
23	0.100	5.010	0.000	0.000	0.058	2.885	0.000	0.000	0.156	7.775	0.000	0.000
24	0.109	5.450	0.000	0.000	0.023	1.170	0.000	0.000	0.098	4.910	0.000	0.000
25	0.100	5.000	0.000	0.000	0.047	2.335	0.000	0.000	0.085	4.260	0.000	0.000
